# SARS-CoV-2-Seroprävalenz bei Kindern und Jugendlichen in Deutschland – ein Überblick

**DOI:** 10.1007/s00103-021-03448-0

**Published:** 2021-11-03

**Authors:** Roma Thamm, Nina Buttmann-Schweiger, Julia Fiebig, Christina Poethko-Müller, Franziska Prütz, Giselle Sarganas, Hannelore Neuhauser

**Affiliations:** Abteilung Epidemiologie und Gesundheitsmonitoring, Robert Koch-Institut, General-Pape-Str. 62–66, 12101 Berlin, Deutschland

**Keywords:** Coronavirus, Antikörper, Kinder und Jugendliche, Immunstatus, Serologie, Coronavirus, Antibodies, Children and adolescents, Immunity, Serology

## Abstract

**Hintergrund:**

SARS-CoV-2-Antikörperstudien ergänzen und erweitern die Erkenntnisse aus der Meldestatistik laborbestätigter COVID-19-Fälle um Informationen zu unentdeckten Fällen.

**Ziel der Arbeit:**

Der vorliegende Beitrag fasst bisherige Ergebnisse zur SARS-CoV-2-Prävalenz aus seroepidemiologischen Studien in Deutschland zusammen, die sich auf Kinder und Jugendliche konzentrieren, und ergänzt die bereits vorliegende Übersicht zur Seroprävalenz bei Erwachsenen und speziell bei Blutspendenden in Deutschland.

**Material und Methoden:**

Die Ergebnisse der Übersichtsarbeit beruhen auf einer fortlaufenden systematischen Recherche in Studienregistern, Literaturdatenbanken, von Preprint-Veröffentlichungen und Medienberichten seroepidemiologischer Studien in Deutschland sowie deren Ergebnissen.

**Ergebnisse:**

Mit Stand 17.09.2021 sind uns 16 deutsche seroepidemiologische Studien, die sich auf Kinder und Jugendliche konzentrieren, bekannt geworden. Für 9 dieser Studien liegen Ergebnisse vor. Für fast alle untersuchten Settings lag die SARS-CoV-2-Seroprävalenz für Kinder im Kita- und Grundschulalter in der ersten COVID-19-Welle deutlich unter 1 % und für Jugendliche unter 2 %. Im Verlauf der Pandemie wurden höhere Seroprävalenzen von bis zu 8 % für Kinder im Grundschulalter ermittelt.

**Diskussion:**

Ergebnisse von SARS-CoV-2-Antikörperstudien bei Kindern und Jugendlichen in Deutschland liegen bislang erst in geringem Umfang und basierend auf lokal-regionalen, nichtrepräsentativen Stichproben vor. In künftigen Studien gilt es, einerseits abzuschätzen, welcher Anteil der Kinder und Jugendlichen bereits eine Infektion hatte oder geimpft ist. Zum anderen gilt es, die Verbreitung körperlicher und psychischer Beeinträchtigungen im Nachgang einer Infektion zu untersuchen.

## Abstract (en)

**Background:**

SARS-CoV‑2 serologic studies complement and expand findings from confirmed COVID-19 cases through identification of undetected cases.

**Objectives:**

This article summarizes previous results on SARS-CoV‑2 prevalence from seroepidemiological studies in Germany focusing on children and adolescents and complements the already existing overview on seroprevalence in adults from general population samples and especially blood donors in Germany.

**Methods:**

The results are based on an ongoing systematic search in study registries, in literature databases, of preprint publications, and of media reports of seroepidemiological studies in Germany and their results.

**Results:**

As of 17 September 2021, we are aware of 16 German seroepidemiological studies focusing on children and adolescents. Results are available for nine of these studies. For almost all settings studied, SARS-CoV‑2 seroprevalence was well below 1% for preschool and elementary school children in the first COVID-19 wave and below 2% for adolescents. As the pandemic progressed, higher seroprevalences of up to 8% were found in elementary school children.

**Discussion:**

Results of SARS-CoV‑2 antibody studies in children and adolescents in Germany are scarce so far and are based on non-representative samples at local or regional level. In future studies, it is necessary on the one hand to estimate which proportion of children and adolescents has already either had an infection or has been vaccinated. On the other hand, it is important to investigate physical and mental health impairments that occur after an infection.

## Einleitung

Am 27.01.2020 wurde die erste bestätigte Infektion mit dem neuartigen Coronavirus SARS-CoV‑2 in Deutschland gemeldet. National und international hat sich das Coronavirus bis dato epidemisch verbreitet und weltweit die COVID-19-Pandemie ausgelöst. Der Nachweis der Virusinfektion erfolgt entweder als direkter Erregernachweis über die Identifikation von Viruserbmaterial bzw. durch Nachweis von Antigen, d. h. viralem Protein, oder indirekt über den Nachweis von spezifischen Antikörpern im Blut, die das Immunsystem im Rahmen der Immunantwort zur Virusabwehr bildet. Wegen der Neuartigkeit des Virus mussten bereits etablierte Instrumente wie das der Echtzeit-Polymerase-Kettenreaktion (RT-PCR) zum Nachweis von Nukleinsäuren (DNA oder RNA) sowie auch Antikörpertests zunächst angepasst oder neu entwickelt werden, was jedoch schon innerhalb der ersten 2 Monate des Jahres 2020 erstmals gelang und seitdem, auch angesichts neuer Virusvarianten, ständig weiterentwickelt wurde [1–3].

Der Einsatz direkter und indirekter Nachweismethoden von SARS-CoV-2-Infektionen ist im Hinblick auf deren Verbreitung von enormer Bedeutung, da diese sowohl symptomatisch als COVID-19-Erkrankung als auch asymptomatisch ohne merkliches Krankheitsgefühl verlaufen können. Jegliche Verläufe sind jedoch einerseits mit Infektiosität, andererseits aber auch mit einer zumindest über einige Zeit andauernden Immunität verbunden. Über alle Altersgruppen hinweg sind valide Daten zur Gesamtheit der Infizierten die zentrale Grundlage zur Einschätzung der Lage und darauf aufbauend der Umsetzung geeigneter Schutzmaßnahmen. Serologische Untersuchungen auf SARS-CoV-2-Antikörper ergänzen die Meldestatistik laborbestätigter Fälle um Informationen zum Anteil der unerkannten Fälle. Dadurch ergibt sich die Untererfassung oder Dunkelziffer. Unter Hinzunahme serologischer Ergebnisse kann zudem der Anteil Verstorbener unter den jemals Erkrankten (Infection Fatality Rate, IFR) genauer bestimmt werden, unter Einschluss auch nichtgemeldeter und asymptomatischer Infektionen.

Asymptomatische oder nur mild symptomatische SARS-CoV-2-Infektionen kommen bei Kindern und Jugendlichen deutlich häufiger vor als bei Erwachsenen [4–8]. Vor diesem Hintergrund sind seroepidemiologische Untersuchungen gerade bei Kindern und Jugendlichen hoch relevant. Wichtig sind solche Studien auch angesichts aktueller Untersuchungen, die darauf hinweisen, dass selbst asymptomatische SARS-CoV-2-Infektionen zu ernst zu nehmenden gesundheitlichen Folgen wie dem pädiatrischen Multiorgan-Immunsyndrom (PMIS) führen können [9, 10]. Der vorliegende Beitrag fasst bisherige Ergebnisse zur SARS-CoV-2-Prävalenz aus seroepidemiologischen Studien in Deutschland zusammen, die sich auf Kinder und Jugendliche konzentrieren. Er ergänzt die bereits vorliegende Übersicht von Ergebnissen zur Seroprävalenz in Zufallsstichproben der Allgemeinbevölkerung mit Fokus auf Erwachsene und bei Blutspendenden in Deutschland [11].

## Material und Methoden

Seit dem Frühjahr 2020 wird im Robert Koch-Institut eine fortlaufende systematische Recherche von seroepidemiologischen Studien in Deutschland sowie deren Ergebnissen durchgeführt und der Kontakt zu den jeweiligen Studienleitungen hergestellt. Die Recherche umfasst Studienregister, Literaturdatenbanken, Preprint-Veröffentlichungen und Medienberichte. Eine ausführliche Beschreibung dieser Recherche inklusive der Suchstrategie und Erläuterungen zu methodischen Unterscheidungsmerkmalen seroepidemiologischer Studien in Bezug auf Stichprobenrahmen und Gütekriterien der eingesetzten Antikörpertests ist im *Journal of Health Monitoring* publiziert [12]. Aus der Recherchetätigkeit resultierte eine seit dem 01.07.2020 verfügbare Webseite in deutscher^1^ und englischer^2^ Sprache. Die regelmäßig aktualisierte Webseite führt Angaben zum Studiendesign sowie Links zu veröffentlichten Studienprotokollen, Studienwebseiten und Ergebnismitteilungen bzw. Publikationen auf. Die Übersicht gruppiert mit Stand vom 01.09.2021 in Antikörperstudien in der Allgemeinbevölkerung (inklusive Studien in bestehenden Kohorten und bei Blutspendenden) und in Studien in besonderen Bevölkerungsgruppen, z. B. bei Beschäftigten in Krankenhäusern, in Betreuungseinrichtungen oder anderen Betrieben. Zu den Studien, die sowohl Erkenntnisse über Antikörper in der Allgemeinbevölkerung als auch in besonderen Bevölkerungsgruppen gewinnen wollen, zählen vor allem Untersuchungen in Kitas, Kindergärten oder Schulen, bei denen sowohl die Kinder oder Jugendlichen als auch die Beschäftigten der Betreuungseinrichtung auf Antikörper getestet werden. Eine interaktive Deutschlandkarte zeigt, wo in Deutschland seroepidemiologische Studien durchgeführt werden.

Für belastbare Aussagen zur Seroprävalenz bei Kindern und Jugendlichen in verschiedenen Altersgruppen reicht die Einbeziehung von Kindern und Jugendlichen in Studien mit Stichproben der Allgemeinbevölkerung oft nicht aus. Daher wurden bisher zumeist Studien bei Kitakindern sowie Schülerinnen und Schülern durchgeführt, aber auch bei Kindern und Jugendlichen, die Vorsorgeuntersuchungen in Kinderarztpraxen in Anspruch nehmen oder die in Kinderkliniken behandelt wurden. Im vorliegenden Beitrag werden Metadaten der einzelnen Studien, wie beispielsweise Studienort oder -region und untersuchte Stichprobe, sowie ihre Ergebnisse tabellarisch dargestellt.

## Ergebnisse

**Figure 1:**
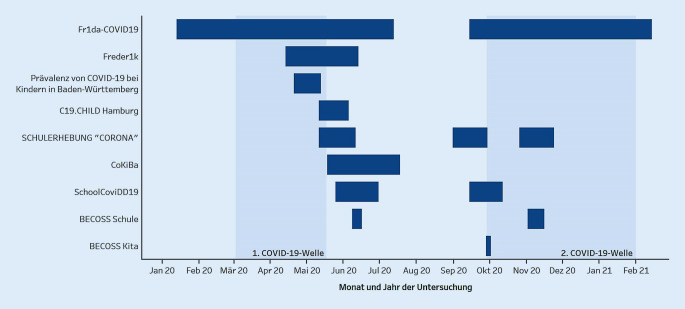

Mit Stand 17.09.2021 haben wir 16 deutsche seroepidemiologische Studien, die sich auf Kinder und Jugendliche konzentrieren, gefunden. Für 9 dieser Studien liegen Ergebnisse vor, die als Studienbericht (1), Pressemitteilung (1), Preprint-Publikation (1) oder als bereits peer-reviewte Publikation (6) veröffentlicht wurden. In Abb. 1 sind die einzelnen Untersuchungszeiträume dieser 9 seroepidemiologischen Studien dargestellt. Um die Studien leichter im Verlauf des Pandemiegeschehens in Deutschland verorten zu können, wurden die Zeiträume der ersten und zweiten COVID-19-Welle zusätzlich visualisiert [13].

Bereits im Januar 2020 startete mit der Fr1da-COVID19-Studie die erste Studie zur Ermittlung der SARS-CoV-2-Seroprävalenz bei Kindern in Deutschland [14]. Diese konnte vor allem deshalb so schnell realisiert werden, weil die Stichprobe auf einer bereits bestehenden Kohortenstudieninfrastruktur basiert, und zwar der Fr1da-Studie in Bayern zur Früherkennung des Typ-1-Diabetesrisikos bei Kindern, die Vorsorgeuntersuchungen wahrnehmen. Ab April 2020 wurden Antikörpertests auch bei einer Unterstichprobe der Freder1k-Studie in Bayern, dem Pendant zur Fr1da-Studie bei Neugeborenen, eingesetzt [15]. Über den Zeitraum Mai bis September 2020 folgten weitere 7 Studien, zum Teil mit Nachuntersuchungen, in verschiedenen Regionen Deutschlands [16–23]. Diese Studien wurden zumeist auf der Basis von Freiwilligenstichproben konzipiert, auch bekannt als Nichtzufallsstichproben oder Convenience Samples.

In den Tab. 1 und 2 sind Metadaten der seroepidemiologischen Studien mit Stichproben gelistet, die auf der Teilnahme an Vorsorgeuntersuchungen, beziehungsweise auf Freiwilligenstichproben in verschiedenen Settings basieren. Die Metadaten beinhalten Angaben zum Studienort oder zur Studienregion, zum Stichprobenrahmen, zum Untersuchungszeitraum, zur untersuchten Stichprobe (Anzahl Teilnehmende und Altersbereich) und zu den eingesetzten Antikörpertests. Für jede Studie sind die Ergebnisse zur berichteten SARS-CoV-2-Seroprävalenz mit 95 %-Konfidenzintervall (KI) angegeben. Für die meisten Studien sind neben der Gesamt-SARS-CoV-2-Seroprävalenz auch stratifizierte Seroprävalenzen für mehrere Altersgruppen angegeben.

**Table 1:** 

Studie	Stichprobenrahmen	Antikörpertest	Zeitraum	Untersuchte Stichprobe	Alter in Jahren	Seroprävalenz (95 %-KI)
*Fr1da-COVID19:* Messung der Entwicklung von Immunität gegen SARS-CoV‑2 in einer Kinderkohorte (Fr1da plus: Typ-1-Diabetesrisiko: Früh erkennen – Früh gut behandeln) in Bayern (2019–2022; [14])	Einladung aller Kinder, die am Diabetes-Typ-1-Screeningprogramm teilgenommen haben, das von hausärztlichen KinderärztInnen während der Vorsorgeuntersuchungen angeboten wurde	Dualer (RBD Anti‑S, Anti-N) LIPS (nicht kommerziell)	01/20–08/20	15.523 Kinder	1–10	0,7 % (0,6–0,8)
			09/20–02/21	11.380 Kinder	1–10	3,9 % (3,6–4,3)
			01/21–02/21	11.380 Kinder	1–5	5,6 % (4,7–6,7)
					6–10	8,4 % (6,4–10,9)
*Freder1k-Studie*: Typ-1-Diabetesrisiko früh erkennen ([15]; Bayern)	Einladung aller Kinder, die am Diabetes-Typ-1-Screeningprogramm teilgenommen haben, das von hausärztlichen KinderärztInnen während der Vorsorgeuntersuchungen angeboten wurde	Dualer (RBD Anti‑S, Anti-N) LIPS (nicht kommerziell)	04/20–06/20	1916 Kinder	Neugeborene	0,5 %
*CoKiBa*: Coronavirus bei Kindern in Bayern ([19]; 3 Regionen mit hohem, mittlerem und niedrigem Infektionsgeschehen)	Einladung aller Kinder mit einem Termin zu einer Vorsorgeuntersuchung 2020 in teilnehmenden Kinderarztpraxen	Elecsys Anti‑N Pan-Ig Roche, inhouse ELISA (RBD Anti-S-IgG)	22.05.2020–22.07.2020	1815 Kinder, die 2020 zu einer Vorsorgeuntersuchung in teilnehmenden Kinderarztpraxen angemeldet waren (für die Auswertung nur jüngstes Kind pro Familie)	1–14	Tirschenreuth 7,2 %
						Regensburg 3,1 %
						Oberbayern/Alpen 1,8 %
				1017 Kinder; Freiwilligenstichprobe	1–18	Tirschenreuth 15,9 %
						Regensburg 2,3 %
						Oberbayern/Alpen 7,8 %

*ELISA* Enzyme-linked Immunosorbent Assay, *IgG* Immunglobulin G, *KI* Konfidenzintervall, *LIPS* Luciferase-Immunopräzipitations-Test, *RBD* Rezeptorbindungsdomäne

**Table 2:** 

Studie	Stichprobenrahmen	Antikörpertests	Zeitraum	Untersuchte Stichprobe	Alter in Jahren	Seroprävalenz (95 %-KI)
**Wohnbevölkerung**						
*Prävalenz von COVID-19 bei Kindern in Baden-Württemberg TEIL A *[22]	Elternteil-Kind-Paare ohne laborbestätigte COVID-19-Diagnose, Rekrutierung über Zeitungen und soziale Medien; Einladung in die Unikliniken Freiburg, Heidelberg, Tübingen und Ulm	Euroimmun S1-IgG, Immunofluoreszenstest + ECLIA, ELISA oder inhouse luminex-basierter Assay	22.04.2020–15.05.2020	2482 Kinder (+2482 Elternteile)	1–10	0,6 % (0,3–1,0)
					1–5	0,5 % (0,2–0,9)
					6–10	0,7 % (0,4–1,4)
**Kinderklinik**						
*C19.CHILD Hamburg *[23]	Stationäre und ambulante PatientInnen aller Hamburger Kinderkliniken, der Wohnbevölkerung sowie Teilnehmende anderer laufender Studien	ECLIA Roche und DiaSorin	11.05.2020–05.06.2020	2436 Kinder und Jugendliche	0–18	1,2–1,5 %
					0–9	1 %
					10–18	2 %
**Schule**						
*SCHULERHEBUNG „CORONA“:* Studie zur Bewertung des Infektionsgeschehens mit SARS-CoV‑2 bei Lehrkräften, Schülerinnen und Schülern in Sachsen ([17]; Leipzig, Dresden, Borna, Werdau, Zwickau)	SchülerInnen und Lehrpersonal aus 18 Schulen in 5 sächsischen Städten	–	05/20–06/20	2344 Kinder und Jugendliche + Erwachsene	7–67	0,6 %
			09/20	2191 Kinder und Jugendliche + Erwachsene	7–67	0,6 %
			11/20	2194 Kinder und Jugendliche + Erwachsene	7–67	1,4 %
					Grundschulkinder^a^	0,5 %
					Jugendliche^a^	1,8 %
*SchoolCoviDD19* ([18]; Ostsachsen)	SchülerInnen der Klassen 8–11 sowie Lehrpersonal aus 13 Schulen	DiaSorin Liaison S1/S2 IgG CLIA + CMIA Abbott (Anti-N), Euroimmun S1-IgG	25.05.2020–30.06.2020	1538 Jugendliche	14–16	0,7 %
				507 Lehrkräfte	37–57	0,2 %
			15.09.2020–13.10.2020	1334 Jugendliche	14–16	0,8 %
				445 Lehrkräfte	36–57	0,2 %
*BECOSS Schule: *Berliner Corona Schulstudie [16, 20]	Jeweils 20 SchülerInnen und 10 LehrerInnen aus 24 Berliner Schulen; Klassen 3–5, 9–11; Schulauswahl über geschichtetes Zufallsverfahren nach Gesundheits- und Sozialstrukturatlas	Euroimmun S1-IgG	11.06.2020–19.06.2020	385 Kinder und Jugendliche (+150 Personal)	8–18	1,3 %
			02.11.2020–16.11.2020	352 Kinder und Jugendliche	9–18	2,0 % (0,8–4,1)
				142 Personal	28–65	1,4 % (0,2–5,0)
				625 Familienangehörige	2–86	1,4 % (0,6–2,7)
**Kita/Vorschule**						
*BECOSS Kita: *Berliner Corona Kitastudie [21]	Jeweils 20 Kinder und 5 Kitamitarbeitende aus 12 Berliner Kitas; Kitaauswahl über geschichtetes Zufallsverfahren nach Gesundheits- und Sozialstrukturatlas	Euroimmun S1-IgG	28.09.2020–02.10.2020	155 Kinder	1–6	0 %
				78 Personal, 487 Familienangehörige	18–78	0,2 %

*KI* Konfidenzintervall, *IgG* Immunglobulin G, *ECLIA* Elektrochemilumineszenz-Immunoassay, *ELISA* Enzyme-linked Immunosorbent Assay, *CLIA* Chemilumineszenz-Immunoassay, *CMIA* Chemilumineszenz-Mikropartikelimmunoassay

^a^ Keine Angabe zur Anzahl und zum Alter

Die Ergebnisse der Studien lassen sich nur schwer übergreifend zusammenfassen. Gründe dafür sind verschiedene Altersbereiche, d. h. Kitaalter (bis ca. 5 Jahre), Grundschulalter (6 bis ca. 10 Jahre) und Jugendalter (11 bis 18 Jahre), eine Rekrutierung in Orten bzw. Regionen mit sehr unterschiedlich hohem Infektionsgeschehen, der meist nicht zufällige Stichprobenzugang mit verschiedenen Selektionseffekten sowie die verschiedenen COVID-19-Wellen (erste Jahreshälfte 2020 und zweite Jahreshälfte 2020 bis Februar 2021). Dennoch lag trotz aller Verschiedenheiten für fast alle untersuchten Settings die SARS-CoV-2-Seroprävalenz für Kinder im Kita- und Grundschulalter in der ersten COVID-19-Welle deutlich unter 1 % und für Jugendliche unter 2 %. Alle seroepidemiologischen Studien, die Nachuntersuchungen beziehungsweise 2. Untersuchungswellen in der zweiten Jahreshälfte 2020 bis Februar 2021 durchgeführt haben, ermittelten höhere SARS-CoV-2-Seroprävalenzen als in der ersten Pandemiewelle. Altersstratifiziert liegen aus der Fr1da-Studie aus Bayern Ergebnisse für Kinder im Kita- und Grundschulalter vor. Für die Gesamtaltersgruppe der 1‑ bis 10-Jährigen ist die Seroprävalenz von 0,7 % auf 3,9 % gestiegen. Am Ende dieser zweiten Beprobungsrunde im Januar–Februar 2021 betrug die Seroprävalenz für Kinder im Kitaalter 5,6 % und für Kinder im Grundschulalter 8,4 %. Für Kinder und Jugendliche berichtet die Berliner BECOSS-Studie einen Anstieg der SARS-CoV-2-Seroprävalenz von Juni 2020 bis November 2020 von 1,3 % auf 2,0 %.

Erst in einer Studie, der Fr1da-Studie, wurde aus der ermittelten Seroprävalenz auf die Untererfassung von SARS-CoV-2-Infektionen bei Kindern geschlossen. Laut Fr1da lag die Untererfassung in der ersten Jahreshälfte 2020 bei Faktor 6 und damit leicht höher als in den im ersten Halbjahr 2020 durchgeführten Studien bei Erwachsenen, in denen eine Untererfassung um den Faktor 4 bis 5 gefunden wurde. Das heißt, die Ergebnisse der Studie legen nahe, dass es etwa 6‑mal so viele SARS-CoV-2-infizierte Kinder gab, wie für diesen Zeitraum nach Infektionsschutzgesetz an die Gesundheitsämter gemeldet wurden. Die Untererfassung sank, den Ergebnissen der Fr1da-Studie nach, in der zweiten Jahreshälfte 2020 bis Anfang 2021 auf den Faktor 3 bis 4 (bei Erwachsenen sank dieser Faktor noch deutlicher auf etwa den Faktor 2; [11, 14]).

## Diskussion

### Seroepidemiologische Studien

Bis Anfang September 2021 (KW 35) sind dem Robert Koch-Institut insgesamt 681.288 laborbestätigte SARS-CoV-2-Fälle für 0‑ bis 19-Jährige übermittelt worden.^3^ Es werden jedoch nicht alle Infektionen labordiagnostisch abgeklärt, wenn sie beispielsweise unbemerkt oder mit milden und unspezifischen Symptomen ablaufen. Da Kinder und Jugendliche sogar häufiger als Erwachsene asymptomatische oder mild symptomatische SARS-CoV-2-Infektionen haben, stellen seroepidemiologische Studien trotz der angesprochenen Limitationen eine essenzielle Datenbasis für die Beurteilung des Infektionsgeschehens dar.

Ergebnisse seroepidemiologischer SARS-CoV-2-Studien in Deutschland, die sich auf Kinder und Jugendliche konzentrieren, liegen bislang erst in geringem Umfang basierend auf lokal-regionalen, nicht repräsentativen Stichproben und nur bis maximal Februar 2021 vor. Hier kommt zum Tragen, dass bei Kindern und Jugendlichen vor der Durchführung von Studien, die mit einer Blutentnahme verbunden sind, eine Reihe von Herausforderungen bewältigt werden müssen, die vom Stichprobenverfahren, der Zustimmung von Ethikkommissionen, der Zulassung von Beprobungsmaterial und Tests bis hin zur Teilnahmebereitschaft von Eltern und Kindern reichen. Die Studien, für die bislang Ergebnisse vorliegen, erlauben in der Gesamtschau noch keine repräsentativen und nach Setting und Altersgruppen belastbaren Aussagen und können zunächst nur grob die Größenordnung des Anteils von bislang mit SARS-CoV‑2 infizierten Kindern und Jugendlichen anzeigen. Diese Größenordnung steht im Einklang mit den Ergebnissen für vergleichbare Zeitpunkte bei Erwachsenen in Deutschland, d. h. mit Seroprävalenzen im niedrigen einstelligen Bereich außerhalb ausgewiesener Hotspots.

### Untererfassung von infizierten Kindern und Jugendlichen

Zur Abschätzung der Untererfassung (Dunkelziffer) ist ein hoher Grad an Repräsentativität notwendig, der bei vielen Stichprobenzugängen der hier dargestellten Studien nicht beurteilbar ist. In der Fr1da-Studie wurde die Untererfassung unter der Annahme berechnet, dass die am Diabetesscreening teilnehmenden, überwiegend jüngeren Kinder bezüglich des Infektionsrisikos der Gesamtheit der Kinder in Deutschland ähnlich sind. Demnach hätte es bei jüngeren Kindern in der ersten Pandemiephase 6‑mal so viele mit SARS-CoV‑2 infizierte Kinder gegeben, wie für diesen Zeitraum nach Infektionsschutzgesetz an die Gesundheitsämter gemeldet wurden, d. h., die Untererfassung wäre ähnlich wie bei Erwachsenen. Allerdings legt der höhere Anteil an asymptomatischen Infektionen bei Kindern im Zusammenhang mit der überwiegend symptombezogenen Testung im Jahr 2020 eine höhere Untererfassung bei Kindern nahe, was auch durch Ergebnisse der TiKoCo-Studie bestätigt wird, die bei Untersuchung einer geschichteten Zufallsstichprobe der Allgemeinbevölkerung ab 14 Jahren des Landkreises Tirschenreuth bis Juli 2020 in der jüngsten Altersgruppe der 14- bis 19-Jährigen die höchste Untererfassung (Faktor 12) berechnet hat [24].

### Vergleich mit weiteren Studien bei Erwachsenen, die Kinder miteinschließen

In der vorliegenden Übersicht nicht enthalten sind die bislang wenigen seroepidemiologischen Studien mit dem Fokus Erwachsene, die auch Kinder und Jugendliche eingeschlossen haben. Die Ergebnisse der KoCo19-Studie in Münchener Haushalten mit Teilnehmenden ab 14 Jahren zeigen für die Altersgruppe der 14- bis 19-Jährigen im Frühjahr 2020 eine SARS-CoV-2-Seroprävalenz von ca. 1,5 %. In der zweiten Runde der Münchener KoCo19-Studie von November 2020 bis Januar 2021 betrug die Seroprävalenz bei 0‑ bis 19-Jährigen 4,3 % (*n* = 212; [25]). Höhere Prävalenzen sind aus den sogenannten Hotspotstudien plausibel, unter denen beispielsweise die TiKoCo-Studie in Tirschenreuth Teilnehmende ab 14 Jahren eingeschlossen hat. Für das Frühjahr 2020 weist TiKoCo eine Seroprävalenz für die Altersgruppe 14–19 Jahre von 10 % aus (*n* = 227 Untersuchte in der Altersgruppe; [24]). Ebenfalls in Tirschenreuth hatte im Sommer 2020 die CoKiBa-Studie eine Seroprävalenz von 7 % bei Jugendlichen und von 5 % bzw. 6 % für Kinder im Kita- und Grundschulalter ermittelt. Wenig überraschend ist, dass die Seroprävalenz noch einmal höher war in der Unterstichprobe der Teilnehmenden, die nicht aufgrund einer Vorsorgeuntersuchung, sondern anlassbezogen die Kinderarztpraxis aufgesucht hatten und getestet wurden [19].

### Vergleich mit internationalen Studien

Auch international sind weit weniger seroepidemiologische SARS-CoV-2-Studien mit dem Fokus Kinder als mit dem Fokus Erwachsene durchgeführt worden [26]. Eine der ersten großen nationalen bevölkerungsbezogenen seroepidemiologischen Studien, die ENE-COVID-Studie aus Spanien, berichtete eine Seroprävalenz von unter 3 % bei Kindern von 1–9 Jahren während der ersten Pandemiewelle [27]. Altersstratifizierte, für die Allgemeinbevölkerung aussagekräftige Seroprävalenzen von SARS-CoV‑2 wurden auch in den Niederlanden während der ersten Pandemiewelle ermittelt. Danach betrug die Seroprävalenz bei Kindern von 2–17 Jahren 1,7 % [28]. Schätzungen für die Prävalenz von SARS-CoV-2-Antikörpern für das Frühjahr 2020 wurden auch für die französische Bevölkerung präsentiert. Die Untersuchung von Restseren im Zeitraum März–Mai 2020 ergab eine landesweite Seroprävalenz bei Kindern unter 10 Jahren von 3 % [29]. Eine Studie aus Dänemark aus dem Sommer 2020 ergab eine Seropositivität für Kinder und Jugendliche von 0–17 Jahren von 1,6 % [30]. Die SEROCoV-POP-Studie vom Frühjahr 2020 im Kanton Genf, Schweiz, berichtete für die Altersgruppe der 5‑ bis 9‑Jährigen eine Seroprävalenz von 0,8 % [31]. In Wien, Österreich, ergab eine von Mai bis Juli 2020 durchgeführte Studie bei Schülerinnen und Schülern (5–21 Jahre) eine SARS-CoV-2-Seroprävalenz von insgesamt 1,3 % [32]. Bei Kindergartenkindern im Alter von 5 Monaten bis 4 Jahren in Frankreich (einschließlich Paris, das zu der Zeit Hochinzidenzgebiet war) wurde eine Seroprävalenz von 4 % gezeigt [33].

Ähnlich wie die CoKiBa-Studie aus Baden-Württemberg im Setting Kinderarztpraxis berichtete auch eine landesweite Studie aus Portugal, die zwischen Mai und Juli 2020 Teilnehmende über ein Netzwerk von Laboratorien für klinische Pathologie und durch öffentliche Krankenhäuser rekrutierte, höhere SARS-CoV-2-Seroprävalenzen von 8 % und 10 % für die Altersgruppen 1–9 Jahre beziehungsweise 10–19 Jahre [34]. Eine ähnliche Gesamtseroprävalenz von 9,5 % berichtete eine von Juli–Oktober 2020 durchgeführte Studie aus dem Großraum Washington, USA, bei pädiatrischen Patientinnen und Patienten im Alter von 2 Monaten bis 22 Jahren [35]. In einer Studie bei 0‑ bis 15-Jährigen in 27 Kinderarztpraxen im Großraum Paris, einem Gebiet, das stark von COVID-19 betroffen war, betrug nach dem Höhepunkt der ersten Pandemiewelle die Rate der Seropositivität 10,7 % [36]. In der RAPID-19-Studie bei 2‑ bis 15-jährigen Kindern von Gesundheitspersonal in 5 Regionen im Vereinigten Königreich (London, Belfast, Cardiff, Manchester, Glasgow) lag die Seroprävalenz für April–Juli 2020 bei 7 % [37].

Für die 2. Pandemiewelle lassen sich national wie international höhere Prävalenzen von SARS-CoV-2-Antikörpern feststellen. Eine Studie bei Kindern und Jugendlichen < 18 Jahren im Bundesstaat Mississippi, USA, ermittelte eine Seropositivität von 2,5 % im Mai 2020 und 16,3 % im September 2020 [38]. Eine Studie aus Belgien im Herbst 2020 stellte für 6‑ bis 15-Jährige eine Seroprävalenz von 4 % in einer Region mit niedriger Transmission und 14 % in einer Region mit hoher Transmission fest [39]. Die 2. Untersuchungswelle der SEROCoV-POP-Studie aus Genf (November–Dezember 2020) ermittelte eine Seroprävalenz bei Kindern im Alter von 0–5 Jahren von 15 % [40]. Auch die Schweizer Studie „Ciao Corona“ bei Kindern im Alter von 6–16 Jahren der Klassen 1–2, 4–5 und 7–8 aus 55 zufällig ausgewählten Schulen in der Region Zürich wurde als longitudinale Untersuchung durchgeführt. Vor dem Hintergrund, dass die Schulen in der Schweiz seit Beginn des neuen Schuljahres im August 2020 bis Jahresende nicht geschlossen wurden, lag die Gesamt-SARS-CoV-2-Seroprävalenz im Sommer bei 2,4 % und im Spätherbst bei 4,5 % der zuvor nicht seropositiven Kinder, was zu geschätzten 7,8 % jemals seropositiven Kindern führte [41].

## Ausblick

Seroepidemiologische Studien zu SARS-CoV‑2 bei Kindern werden auch im weiteren Verlauf der Pandemie von Interesse sein. Es gilt dabei, zum einen abzuschätzen, welcher Anteil der Kinder und Jugendlichen bereits eine Infektion hatte oder geimpft ist. Zum anderen gilt es, die Verbreitung körperlicher und psychischer Beeinträchtigungen im Nachgang einer Infektion zu untersuchen. Um Ergebnisse solcher Studien auf unselektierte Kinder und Jugendliche in Deutschland übertragen zu können, ist eine weitere Optimierung der Stichprobenzugänge anzustreben. Dies gilt insbesondere für den Stichprobenzugang Vorsorgeuntersuchung, da beispielsweise Kinder und Jugendliche aus Familien mit niedrigem Sozialstatus seltener an Früherkennungsuntersuchungen teilnehmen als Gleichaltrige aus Familien mit hohem Sozialstatus [42].

Die Unterscheidung von Antikörpern nach Infektion und nach Impfung ist prinzipiell möglich, hat aber Limitationen. Gegen das Spike-Protein (Anti-S) gerichtete Antikörper sind sowohl nach einer Infektion als auch nach einer Impfung nachzuweisen, während Antikörper gegen das Nukleokapsid-Protein (Anti-N) sich nur nach einer Infektion mit SARS-CoV‑2 entwickeln. Allerdings sind Antikörper keine über die Zeit konstanten Marker einer stattgehabten Infektion. Außerdem unterscheiden sich Verläufe von Antikörperspiegeln im Serum je nach Zielstruktur der Antikörper (Anti‑S, Rezeptorbindungsdomäne [RBD] oder Anti‑N; [43, 44]). Sowohl initial als auch im weiteren zeitlichen Verlauf nach einer Infektion weichen Antikörpertests verschiedener Hersteller zum Teil sehr deutlich in ihren Testgütekriterien, d. h. ihrer Sensitivität und Spezifität, voneinander ab [45, 46]. Zudem steigen die Antikörperspiegel nach einer asymptomatischen Infektion oder einem milden Krankheitsverlauf schon initial weniger hoch an als bei mittleren oder schweren Krankheitsverläufen [47, 48]. Hinzu kommt das Phänomen des sogenannten Waning bis hin zur Seroreversion, also dem im Zeitverlauf nach einer Infektion (oder Impfung) fortschreitenden Abfall von Antikörperspiegeln unter den Testgrenzwert [49]. Eine Adjustierung für Waning und Seroreversion wird mit länger werdenden Zeiträumen zwischen Studienfeldphase und Zeitpunkt der Infektion immer bedeutsamer werden [50].

Ergebnisse weiterer seroepidemiologischer Studien mit Fokus Kinder und Jugendliche in Deutschland werden in Kürze erwartet. Geplante Studien, die durch Pressemitteilungen, Registrierung von Studienprotokollen oder über Netzwerke und persönliche Mitteilungen bekannt werden, werden zeitnah in die fortlaufende Übersicht auf der Webseite des Robert Koch-Instituts aufgenommen. Dies ermöglicht die weitere Vernetzung, den Austausch und die zeitnahe Aktualisierung der hier vorliegenden Ergebnisübersicht.

## Footnotes

1 www.rki.de/covid-19-ak-studien.

2 www.rki.de/covid-19-serostudies-germany.

3 http://www.rki.de/covid-19-altersverteilung.
